# Editorial: Salinity Tolerance in Plants: Mechanisms and Regulation of Ion Transport

**DOI:** 10.3389/fpls.2017.01795

**Published:** 2017-10-24

**Authors:** Vadim Volkov, Mary J. Beilby

**Affiliations:** ^1^Faculty of Life Sciences and Computing, London Metropolitan University, London, United Kingdom; ^2^Department of Plant Sciences, College of Agricultural and Environmental Sciences, University of California, Davis, Davis, CA, United States; ^3^School of Physics, University of New South Wales, Sydney, NSW, Australia

**Keywords:** salinity tolerance, halophytes, ion channels, ion transporters, systems biology, synthetic biology, salt glands, halotropism

Life presumably arose in the primeval oceans (with similar or even greater salinity than present ocean—Knauth, 1998), so the ancient cells were designed to withstand salinity. However, for the plants to “land,” their immediate ancestors most likely lived in fresh, or slightly brackish, water. These algae already developed roots/rhizoids to obtain nutrients from the substrate and did not face hyper-salinity, as well as air exposure, in a drying pond (Raven and Edwards, 2001; Flowers et al., 2010). The fresh/brackish water origins might explain why many land plants, including some cereals, can withstand moderate salinity, but only 1–2% of all the higher plant species survive at salinities close to that of seawater (Santos et al., 2016). Salt tolerance does not have a firm dividing line: 80 mM NaCl is usually taken as a stress threshold and 200 mM NaCl as the halophyte territory (compare to ~470 mM NaCl content of the modern seawater). However, plants have re-discovered their saline origins in more than 70 independent halophytic lines (Bennett et al., 2013).

Salinity is among the major threats to agriculture, having been one of the reasons for the demise of the ancient Mesopotamian Sumer civilization (Jacobsen and Adams, 1958) and in the present time causing huge annual economic losses of over 10 billion USD (this figure exceeds the gross domestic product of more than 50 less-developed countries of the modern world—Qadir et al., 2014). The effects of salinity on plants include osmotic stress, disruption of membrane ion transport, direct toxicity of high cytoplasmic concentrations of sodium and chloride on cellular processes and induced oxidative stress. Ion transport is the crucial starting point that determines salinity tolerance in plants: this includes the cation and anion transport across the plasma membranes of the root cells, the transport through the vacuolar membranes, the long-distance ion transport via xylem and phloem and the salt excretion and accumulation by the specialized cells.

Transport via membranes is mediated mostly by the ion channels and transporters, which ensure selective passage of specific ions. In the model salt-sensitive plant, *Arabidopsis thaliana*, over a thousand genes are predicted to encode membrane proteins: over one hundred of these determine the cation channels and transporters (Mäser et al., 2001). The molecular and structural diversity of these ion channels and transporters is amazing. They differ in the number of protein transmembrane domains, in the selectivity filters for allowing passage of specific ions, in the molecular structures for gating (opening and closing) by the change of trans-membrane potential difference or by chemical compounds and also in regulation by the interacting proteins or the chemical modifications (e.g., by phosphorylation and dephosphorylation, SUMOylation, S-nitrosylation, potentially carbamylation etc.).

Naturally occurring salt-tolerant plants, halophytes, which survive at high salt concentrations, provide a unique source of traits for the tolerance and of genes for membrane proteins involved in ion transport and their regulators: the genes that are functioning under salinity and could be transferred to agriculturally important crops to increase their tolerance. The older lineage of Chlorophyta contains many marine algae with ion transporters, channels and pumps, potentially unknown in land plants. Fungi can also provide useful transporters, not found in Kingdom Plantae. An alternative approach, drawn from synthetic biology, is to modify the existing membrane transport proteins or to create new ones, with the desired properties, for transforming of the agricultural crops. Obtaining the detailed descriptions of distinct ion channels and transporters present in halophytes, and then progressing to the cellular and the whole plant mechanisms, is the logical way to understand salinity tolerance. The theoretical scientific approaches involve protein chemistry, structure-function relations of membrane proteins, systems biology and physiology of stress and ion homeostasis.

At the time of compiling this Research Topic many aspects of ion transport under salinity stress are not yet well understood. The structure-function relationships of ion channels and transporters are slowly being deciphered, but essentially remain *terra incognita*. The multiple links between the ion fluxes, electrophysiology and other physiological processes, leading to salinity tolerance, are often not described in any detail. Completely unexpected features of ion transport under salinity stress may be waiting to be discovered. The Research Topic has attracted researchers in ion transport and salinity tolerance of plants (and fungi) and we have combined our efforts to achieve a wider, more detailed understanding of salt tolerance in plants mediated by ion transport.

The papers in the Research Topic address the aspects of ion transport under salinity stress mentioned above: they analyse precise details of the molecular structure of ion transporters linked to the selectivity of ion transport and compare ion fluxes in different organisms. The results suggest a diversity of adaptations to salt stress and ion transport in yeast and other fungi, in algae, in agriculturally important plants and crops and further in halophytes from mangroves to recretohalophytes, which excrete salt via specialized salt glands. The topic contributions explore numerous aspects of salinity tolerance, including osmotic adjustment, oxidative stress, changes in photosynthesis and morphology of cells. The wide and comprehensive picture of the processes related to salinity tolerance is united under the heading of ion transport at the time of saline stress. The following short introduction, describing the contributing papers, provides a brief guide to the topics that have been addressed.

The reviews in this topic lay out a logical pathway for understanding the ion transport and physiology of plants affected by salinity: from characterization of Na^+^ fluxes and their regulation at the level of the whole plant to fluxes at the cellular and vacuolar membranes with specific ion channels and transporters. Maathuis et al. provide a solid description of the morphological structures and the molecular mechanisms important for Na^+^ transport. Volkov applies a quantitative approach to ion transport, initially in yeast and other small cells and then to entire plants (Volkov; Volkov). The current advanced methods allow us to estimate the exact number of the individual ion channels and transporters in a single yeast cell. Further, we can determine their uneven distribution in specific lipid domains with a size around hundreds of nanometers. The ion fluxes via cellular membranes are also influenced by the cell walls and by the hydrostatic pressure inside cells, adding more complexity (Volkov). A similar quantitative approach is useful for the whole plants, although much less is known about the larger organisms. In a second review, Volkov applies a systems biology approach, describing the progress in genetic and protein engineering to manipulate ion fluxes in plants (Volkov).

The Characeae are one of the three charophyte branches of the phylogenetic tree that gave rise to land plants. Beilby provides a comprehensive review of salinity tolerance in giant-celled algae of the Characeae, introducing this classical system and its response to salinity, measurement of ion fluxes and study of fast electric action potentials. The narrative compares the salt-tolerant *Chara longifolia* and *Lamprothamnium* species with the salt-sensitive *Chara australis* and discusses several electrically active states of the plasma membrane, when different sets of ion transporters determine the membrane electrophysiology. The review ponders both hyper and hypo-osmotic regulation in these unusually large cells (Beilby).

Moving to more saline environments in a brief perspective paper on halophytes, Duarte et al. focus on morphological features of halophytes, particularly photosynthesis, accumulation of osmotically active substances and antioxidants protecting the plant exposed to high salinity. Yuan et al. and Dassanayake and Larkin present reviews that discuss novel advances in research on the ion transport via salt glands of excreting salt recretohalophytes. The former review focuses on the morphological structure of salt glands, methods of collection of salt gland liquid, mechanisms of ion transport including the specific ion channels and transporters involved (Yuan et al.). The latter review discusses morphology and evolution of salt glands, suggesting that the salt glands emerged independently at least 12 times in recretohalophytes. Further narrative briefly describes the present genetic resources available for genetic engineering of salt glands and ponders the strategies and complications on the way (Dassanayake and Larkin).

Research papers within the Topic feature two reports analysing macroscopic results of single amino acid substitutions within specific regions of potassium HAK5 (Alemán et al.) and cation HKT (Almeida et al.) transporters. The plant transporter HAK5 is important for K^+^ uptake at low K^+^ concentrations in the soil. The mutation F130S in HAK5 from *A. thaliana* substituted phenylalanine to serine in a presumed pore for K^+^ binding of the transporter. The mutated HAK5 increased the survival of yeast cells of strain 9.3 under salinity. These yeast cells lacked their own K^+^ and Na^+^ transport systems, but expressed the mutated HAK5 from *Arabidopsis* (Alemán et al.). The kinetic transport properties of mutated HAK5 were also characterized in the yeast 9.3 cells. The F130S mutation surprisingly increased affinity for Rb^+^ (Rb^+^ mimicked K^+^ uptake) over 100 times, while reducing the inhibitory constants K_i_ of the Rb^+^ transport by Na^+^ or by Cs^+^ over 10 times. Several other HAK5 mutants also demonstrated altered kinetic properties of transport (Alemán et al.). The HKT cation transporters play a role in Na^+^ and K^+^ transport to xylem and in uptake of the ions by roots. A single amino acid change from serine to glycine (S70G) in the first pore domain of tomato HKT1;2 (*Sl*HKT1;2) transporter altered the ion selectivity of transport. The mutated form of *Sl*HKT1;2 transported K^+^ and Na^+^, instead of only Na^+^, but at lower rates. The selectivity of ion transport was characterized when *Sl*HKT1;2 was expressed in oocytes of African clawed toad *Xenopus laevis*. The ion currents were measured by the electrophysiological method of two-electrode voltage clamp. The heterologous expression of tomato HKT1;2 in the mutant *Arabidopsis athkt1;1* plants without native HKT transporter restored the K^+^ accumulation in the shoots. Thus the combined methods of molecular biology and electrophysiology characterized the functioning of *Sl*HKT1;2 transporter in tomato (Almeida et al.).

Two more experimental papers link expression of the individual genes with salinity tolerance and ion transport (Liu et al.; Jing et al.). In barley under salinity treatment the expression of slow anion channel genes, *HvSLAH1* and *HvSLAC1*, was up-regulated in the leaves of salt-tolerant cultivars and positively correlated with the grain yield under field conditions. Compared to sensitive varieties, the aperture of stomatal cells under salinity remained larger in the salt tolerant barley plants, although significantly decreased in comparison to control conditions (Liu et al.). The trees of the mangrove *Kandelia candel* grow at the sea coast with roots in seawater and the expression of its superoxide dismutase gene *KcSOD* in tobacco plants increased their salt tolerance (Jing et al.). Further, the Na^+^ fluxes in roots, the accumulation of ions, the activity and intracellular location of superoxide dismutase, the kinetics of reactive oxygen species and the parameters of photosynthesis or growth were characterized for the mangrove, the transgenic tobacco and the *Arabidopsis* plants (Jing et al.).

Three experimental papers report important physiological aspects of ion transport and salinity tolerance for different plant species. The sodium accumulation in vacuoles and in the cytoplasm of distinct root zones was explored in detail for six salt-tolerant and sensitive wheat cultivars by using a sodium-sensitive fluorescent dye. Unexpectedly, the cells of root meristem in the salt-tolerant cultivars had a higher Na^+^ in their cytoplasm than those of the salt-sensitive cultivars (Wu et al.). The responses to salinity of salt-tolerant and salt-sensitive varieties of habanero pepper plants (*Capsicum chinense*) also differed greatly (Bojórquez-Quintal et al.). The salt tolerant variety accumulated fifty times more osmolyte proline in roots, retained more K^+^ in the roots under salinity, sequestered Na^+^ in the cytoplasm in vacuole-like structures and not in the apoplast, compared to the salt sensitive pepper plants (Bojórquez-Quintal et al.). The halophyte *Atriplex canescens* (fourwing saltbush) increased the net photosynthetic rate, accumulated more proline and betaine, kept relatively stable K^+^ concentrations in its tissues and had higher Na^+^ in the salt glands under salinity than under non-saline conditions (Pan et al.).

Finally, three other experimental papers elucidate several novel areas, usually not associated with salt stress. Selected strain BG03 of the soil bacterium *Bacillus subtilis* improved salinity tolerance of the white clover by decreasing the osmotic potential of the leaves and preserving better K^+^/Na^+^ ratio under salinity (Han et al.). The examination of potato plantlets, using electron microscopy, revealed unusual features of the ultrastructure of leaf mesophyll cells and their chloroplasts, which were recorded under a gradient of salinity treatments (Gao et al.). The associated biochemical tests and X-ray microanalysis of plant tissues added more details to understanding the survival of these plantlets under saline conditions (Gao et al.). The salt avoidance bending of growing roots, known as halotropism, was documented for *Arabidopsis* plants (Yokawa et al.). Halotropism was influenced by the illumination stress for these roots. The illumination stress increased their growth rate, stimulated oxidative stress and influenced F-actin-dependent processes. The UVR8 light receptor relocated to nuclei in apical root cells after UV-B treatment, while the halotropism was inhibited by light (Yokawa et al.).

We believe that our simultaneous efforts will inspire further research and wider understanding of the ion transport in general and at the time of salinity stress. We also hope that the Research Topic lays one more foundation stone in understanding the biophysics of ion transport via ion channels and transporters *en route* to deciphering salinity tolerance, creation of salt-tolerant crops, and of growing crops in the fields irrigated with salt water.

## Author contributions

All authors listed have made a substantial, direct and intellectual contribution to the work, and approved it for publication.

### Conflict of interest statement

The authors declare that the research was conducted in the absence of any commercial or financial relationships that could be construed as a potential conflict of interest.
